# Effect of preoperative immunonutrition on postoperative short-term clinical outcomes in patients with gastric cancer cachexia: a prospective randomized controlled trial

**DOI:** 10.1186/s12957-024-03348-y

**Published:** 2024-04-17

**Authors:** Junjian Yu, Antai Yuan, Qi Liu, Wei Wang, Yuqi Sun, Zequn Li, Cheng Meng, Yanbing Zhou, Shougen Cao

**Affiliations:** Department of Gastrointestinal Surgery, The Affiliated Hospital of Qingdao University, 16# Jiangsu Road, Qingdao, Shandong Province 266000 P.R. China

**Keywords:** Gastric cancer, Cancer cachexia, Immunonutrition, Enteral nutrition, Preoperative intervention

## Abstract

**Background:**

Although current guidelines(ESPEN guideline: Clinical nutrition in surgery and other guidelines) recommend preoperative immunonutrition for cachectic gastric cancer patients, the strength of the recommendation is weak, and the level of evidence is low. The benefits of preoperative immunonutrition still remain controversial.

**Patients and methods:**

112 patients with gastric cancer cachexia were enrolled in the study and randomly assigned in a 1:1 ratio to receive either preoperative enteral immunonutrition support (IN, *n* = 56) or standard enteral nutrition support (SEN, *n* = 56). The primary endpoint was the incidence of infectious complications, and the secondary endpoints included the nutritional indicators, inflammatory markers, immune parameters, postoperative recovery and complications and gastrointestinal intolerance reactions.

**Results:**

The incidence of postoperative infectious complications(*P* = 0.040) and overall complications (*P* = 0.049)was significantly lower in the IN group compared to the SEN group. In terms of laboratory inflammatory indexes, patients in the IN group demonstrated significantly lower levels of white blood cells (WBC), C-reactive protein (CRP), and interleukin-6 (IL-6), as well as higher levels of lymphocytes (LYMPH) and immunoglobulin A (IgA), compared to patients in the SEN group, with statistically significant differences. In terms of clinical outcomes, the IN group had a shorter duration of antibiotic use (*P* = 0.048), shorter hospital stay (*P* = 0.018), and lower total hospital costs (*P* = 0.034) compared to the SEN group. The IN group also experienced significantly less weight loss after surgery (*P* = 0.043).

**Conclusion:**

Preoperative administration of immunonutrition formula has a positive impact on the incidence of infectious complications in patients with gastric cancer cachexia after surgery. It improves patients’ inflammatory and immune status, shortens hospital stays, and reduces healthcare costs. Preoperative use of immunonutrition may contribute to the improvement of prognosis in this high-risk population.

**Supplementary Information:**

The online version contains supplementary material available at 10.1186/s12957-024-03348-y.

## Introduction

Gastric cancer is the fifth most common cancer worldwide and the third leading cause of cancer deaths globally. In 2020, there were an estimated 1.09 million new cases of gastric cancer and 768,000 deaths globally, according to statistics. The incidence of gastric cancer varies widely between regions, with the highest rates observed in East Asia, particularly in Japan, Korea, and China. Gastric cancer is a deadly disease, and the mortality rate is high [1, 2]. 

Cachexia is a malnutrition-related disorder commonly associated with chronic illnesses and often accompanied by non-specific inflammation, representing a distinct form of malnutrition [3]. In 2011, an international consensus led by Professor Kenneth Fearon provided a definition for cachexia as a multifactorial syndrome characterized by persistent skeletal muscle wasting, with or without concurrent adipose tissue loss, which cannot be fully ameliorated by conventional nutritional interventions and ultimately leads to progressive functional impairment [4]. Cachexia frequently occurs in various chronic diseases, including malignant neoplasms, chronic obstructive pulmonary disease, chronic heart failure, chronic renal failure, hepatic insufficiency, HIV/AIDS, and rheumatoid arthritis, among others. Notably, cancer cachexia has a high incidence and represents a common complication in advanced malignant tumors [5]. Cancer cachexia is a continuum that can be roughly categorized into three stages: precachexia, cachexia, and refractory cachexia. Pre-cachexia is often difficult to identify and is commonly overlooked in clinical practice. There is no linear temporal progression between precachexia and refractory cachexia, and emerging consensus suggests that preventing cancer cachexia is more important than treating it [6, 7]. For patients with curatively treated upper gastrointestinal cancer, it is imperative to promptly identify and intervene upon precachexia. Reported data suggests that 60–80% of cancer patients may experience cachexia, which is associated with a progressive decline in functional status, increased cancer-related mortality rates, treatment-related complications, and diminished quality of life [8]. Thus, effective interventions targeting cancer cachexia hold substantial implications for the long-term survival of individuals with cancer.

Unlike simple malnutrition, cachexia is also associated with metabolic abnormalities [9–11]. Dysregulation of tumor-associated genes leads to increased mediators of breakdown metabolism. Additionally, cancer-induced inflammation can generate pro-inflammatory cytokines [12]. The principal metabolic characteristics in patients with cancer cachexia encompass increased energy expenditure, heightened protein and/or fat catabolism, and reduced protein synthesis. These processes likely involve mechanisms such as neuroendocrine hormone dysregulation, inflammation, and inflammatory factors including tumor necrosis factor-alpha (TNF-α) and interleukins (ILs). Additionally, special metabolic factors such as fat mobilizing factors and protein hydrolysis-inducing factors may also play a role [13–15].

Immunonutrition is a type of medical nutrition therapy that involves the use of specialized nutrients to support the immune system and improve clinical outcomes in patients with cancer. It is based on the concept that certain nutrients, such as omega-3 fatty acids, arginine, and nucleotides, have immunomodulatory properties that can enhance the body’s ability to fight cancer and promote healing after surgery or other treatments [16, 17]. The use of immunonutrition in cancer has gained significant interest in recent years, as there is growing evidence to support its potential benefits in improving clinical outcomes in cancer patients. However, due to the small sample size of current studies and insufficient clinical guideline level for immunonutrition [18–20], more research is needed to fully understand its mechanisms of action, monitor patient response to treatment, evaluate its impact on clinical outcomes, determine which patients are most likely to benefit from this therapy, and develop standardized protocols for the use of immunonutrition in cancer patients.

Therefore, the aim of this randomized controlled trial (RCT) is to investigate the impact of preoperative immunonutrition on the postoperative clinical outcomes of gastric cancer cachexia patients.

## Materials and methods

This study is a prospective, parallel-group, open-label, positive-controlled, randomized clinical trial compared the effects of preoperative administration of two different enteral nutrition formulas on patients. This work was conducted in the Department of Gastrointestinal Surgery, Affiliated Hospital of Qingdao University, from 2022 to 2023. The study protocol was approved by the Institutional Review Board of Qingdao University Affiliated Hospital (No. QYFYEC2023-37) and conducted in accordance with the ethical standards of the 1975 Helsinki Declaration. All participants were fully informed about the study protocol and provided written informed consent. The study protocol complied with CONSORT standards and was registered in the Chinese Clinical Trials Registry with registration number ChiCTR230007624.

### Sample size

The sample size calculation for this study was based on historical data and assumptions. Previous studies have shown that the incidence of postoperative infectiouscomplications in gastric cancer cachexia patients is about 33.6%, and the use of immunonutrition support can reduce complications by about 10% [21]. Assuming that the follow-up period is 12 months and the non-inferiority margin is set at 0.15, with a 1:1 random ratio, a significance level of α = 0.025 (one-sided), a power of 1-β = 80%, and an attrition rate of 10% for either group, a minimum of 112 patients are needed for this study.

### Selection of patients

**Fig. 1:**
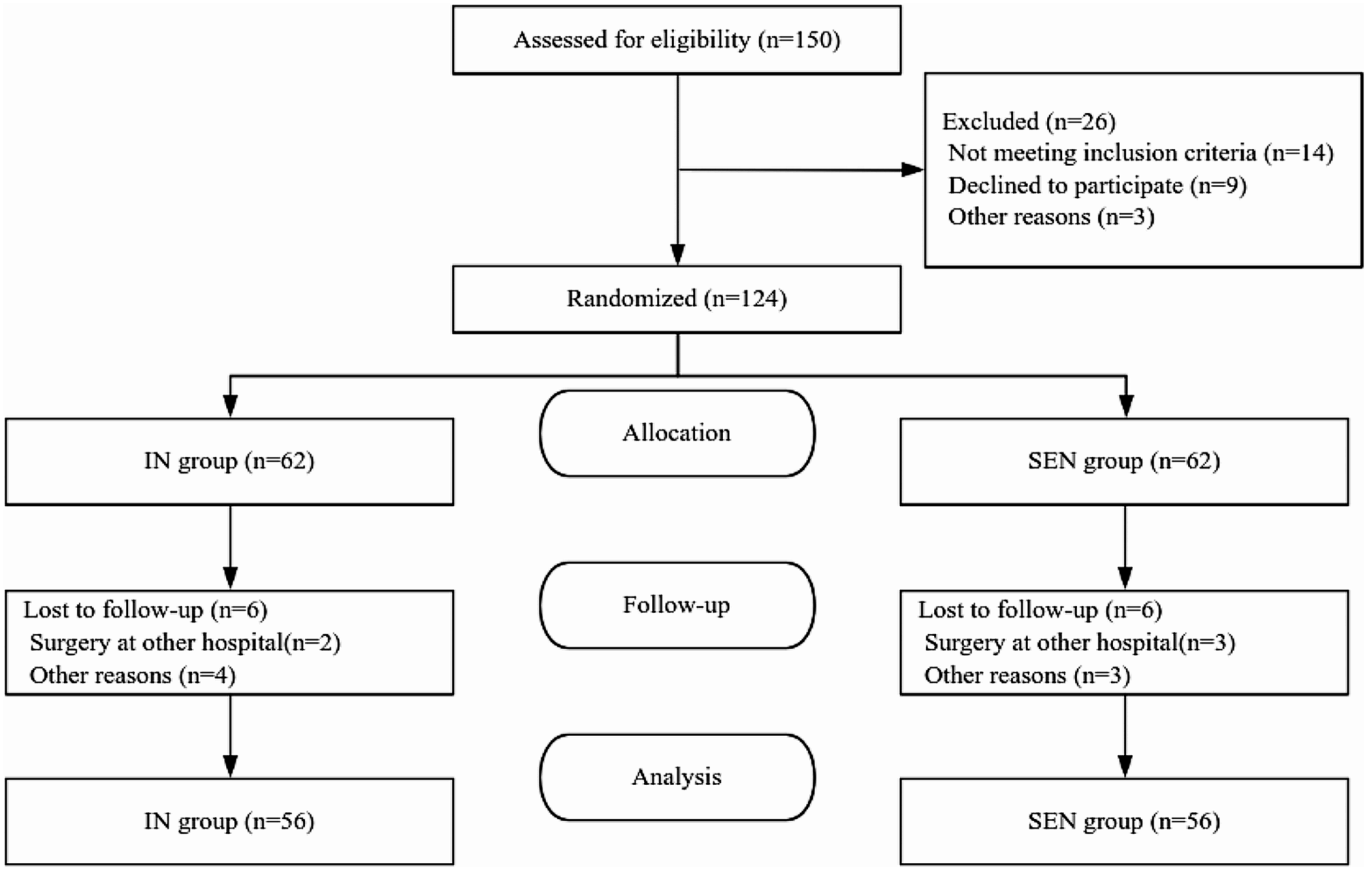
CONSORT Flow Diagram. Study flowchart. SEN, standard enteral nutrition group; IN, immunonutrition group

The present study was conducted at the Department of Gastrointestinal Surgery, Qingdao University Affiliated Hospital, from May 2023 to December 2023. Patients aged 18 years or older, diagnosed with gastric adenocarcinoma, assessed with cancer cachexia, and planned to undergo robot-assisted or laparoscopic radical gastrectomy with sufficient time for nutritional intervention before the surgery were included. The diagnostic criteria for cachexia uses the international consensus established in 2011: Weight loss > 5% over past 6 months; or BMI < 20 and any degree of weight loss > 2%; or appendicular skeletal muscle index consistent with sarcopenia (males < 7.26 kg/m^2^; females < 5.45 kg/m^2^ degree of weight loss > 2% [4].The patient selection process is shown in Fig. 1.

If a participant meets any of the following criteria at baseline visit, they will be excluded from the study: (1) Refusal to participate in the trial; (2) Emergency surgery; (3) Presence of contraindications to enteral nutrition (such as decompensated short bowel syndrome, severe peritonitis, severe gastrointestinal motor disorder, unstable vital signs, coagulation dysfunction, severe nausea, vomiting); (4) Impaired heart/liver/kidney function; (5) Evidence of bacterial infection and/or autoimmune disease currently present; 6.Patients receiving neoadjuvant therapy, tumor immunotherapy or using medications with significant immune modulatory functions (such as PD-1 or PDL-1 inhibitors); 7. History of allergy or intolerance to any component of the trial product; 8. Presence of psychiatric disorders, alcoholism or other conditions that the investigators consider might affect the ingestion of the study product or compliance with the study protocol; 9. Follow-up failure; 10. deviation from the trial protocol; 11.Inability to perform curative gastrectomy during surgery; 12. Occurrence of severe adverse events closely related to the intervention of this trial.

### Randomization and intervention

After excluding patients who did not meet the inclusion criteria, we used SPSS 26.0 (IBM, Armonk, NY, USA) statistical software to conduct randomization through computer-generated sequences. Experimental group (IN group) and Control group (SEN group) were designed.Upon patient enrollment, specialized researchers administer enteral nutrition and provide clear instructions on its usage for intervention. Daily follow-up phone calls are conducted twice a day to supervise patient adherence. Although blinding the patients was not achieved, the surgical doctors, radiologists, pathologists, and data managers remain unaware of the procedures received by the patients.

The IN group received an enteralimmunonutrition supplement containing ω-3 polyunsaturated fatty acids, L-arginine, and nucleotides, providing approximately 1063 kcal(Please refer to supplementary material for the immunonutritional formula). The control group received isonitrogenous and isocaloric standard enteral nutrition,as nutritional support from day − 7 to day − 1 before surgery. In order to reach the targeted energy and protein intakes (25–30 kcal/kg per day and 1.2–1.5 g protein/kg per day), we will provide patients with professional dietary guidance to make up for the energy deficit. Both groups underwent parallel surgical procedures and received similar therapeutic care.

All enrolled patients adhered to the enhanced recovery after surgery (ERAS) program, which is the routine care mode utilized in our center [22]. The ERAS program consists of preoperative pre-rehabilitation therapy [23], no preoperative mechanical bowel preparation, fasting for 6 h before surgery, orally glucose infusion until 2 h before surgery, intraoperative target-oriented liquid management, local anesthesia in the deep incision, general anesthesia combined with epidural anesthesia, early removal of urinary catheter and abdominal drainage tube, early bedside activity, multimodal postoperative analgesia, and sequential enteral nutrition treatment after awakening.

### Surgery, discharge criteria and follow-up

Laparoscopic or da Vinci robot gastrectomy for radical treatment of gastric cancer was performed by the same surgical team that carried out more than 100 cases of gastric cancer radical surgery annually. The surgical procedure followed the Japanese Gastric Cancer Treatment Guidelines 2021 (6th edition) [24].The type of digestive reconstruction depends on the surgeon’s habits, intraoperative status, and past experience. The choice of laparoscopic or da Vinci assisted surgery depends on the patient’s subjective wishes.

The study group conducted three ward inspections per day to check compliance and observe outcomes. After the first bowel movement, the patient could gradually accept liquid and semi-liquid diets. Discharge criteria for this study were as follows: (1) well-controlled postoperative pain scores (visual analogue scale < 4points); (2) oral semi-liquid diet without intravenous fluids; (3) satisfactory exercise program (6 h or more per day); (4) adequate out-of-hospital care; (5) voluntary discharge; (6) no fever, abdominal pain, infection and other surgical complications. Additionally, contact information and address will be confirmed for each patient prior to discharge.After discharge, telephone follow-up twice a week was conducted to determine postoperative complications. Notify the patient to schedule a follow-up outpatient consultation for further assessment and continue the telephone follow-up until 30 days after the surgery.

### Outcomes

The primary outcome of the study was the incidence rate of postoperative infectious complications, defined as bacterial infections occurring within 30 days after surgery. The diagnosis of infectious complications was based on fever (≥ 38℃), elevated C-reactive protein (CRP) levels, specific clinical symptoms of infection, and positive bacterial culture. The diagnostic criteria for postoperative infections in this study were as follows:

- Pneumonia: The imaging examination reveals inflammatory infiltration in the lungs, accompanied by respiratory distress and decreased arterial oxygenation. The patient also exhibits pneumonia-related signs and positive bacterial culture in the sputum. Obtaining sputum culture may be challenging for postoperative patients with weakened coughing ability. Therefore, when typical clinical manifestations and radiographic features of pneumonia are present and other possible causes of infection have been ruled out, a diagnosis of pneumonia can be made even in the absence of microbiological evidence [25].
- Urinary tract infection: Symptoms such as difficulty in urination, leukocyturia, and bacteriuria (with a colony-forming unit count exceeding 10,000 colonies per milliliter) are present, along with positive results in urine culture or urethral secretion culture.
- Abdominal infection: Characterized by abdominal symptoms and positive signs such as abdominal pain, tenderness, rebound tenderness, evidence of imaging (such as intra-abdominal abscess), and positive bacterial culture from intra-abdominal smear or abdominal drainage fluid.
- Catheter-related bloodstream infection: Refer to the guidelines [26].

Secondary outcomes included the following:

- Nutritional indicators: Serum albumin(ALB), prealbumin(PAB), hemoglobin(HB), and weight changes at baseline, preoperatively, and on postoperative days (PODs) 1, 3, and 5.
- Inflammatory markers: Baseline, preoperative, and postoperative levels of white blood cell count (WBC), CRP, procalcitonin (PCT), interleukin-1, 6, 8 (IL-1, 6, 8), interferon-gamma (IFN-gamma), and TNF-α concentration.(The measurement utilizing the ELISA method.)
- Immune parameters: Baseline, preoperative, and postoperative counts of lymphocytes (LYMPH), CD4 + T cells, CD8 + T cells, CD4+/CD8 + ratio, and concentrations of serum immunoglobulin A, MandG.
- Postoperative recovery and complications: Time to first flatus and bowel movement, length of hospital stay(LOS), total hospitalization costs, 30-day readmission rate, 30-day mortality rate, duration of antibiotic use, occurrence of other postoperative complications, and start time of postoperative chemotherapy.
- Gastrointestinal intolerance reactions: Incidence of nausea, vomiting, diarrhea, abdominal pain, bloating, constipation, and other related symptoms.

The definitions of other postoperative complications are as follows:

- Surgical anastomotic leak: drainage of digestive fluid or food via an abdominal drainage tube, and confirmation of anastomotic leak through upper gastrointestinal (GI) radiography.
- Gastroparesis: upper abdominal distension, vomiting, gastric decompression revealing large amounts of gastric contents, and confirmation of delayed gastric emptying through upper gastrointestinal x-ray.

### Statistical analysis

The Shapiro-Wilk test was used to verify normality of quantitative variables. Quantitative variables that follow a normal distribution are described using the mean and standard deviation, while median and interquartile range (IQR) are used for those that do not follow a normal distribution. For categorical variables, frequency and percentage are used. Proportional comparison tests are based on chi-square test or Fisher’s exact test. Quantitative variable analysis is performed using Student’s t-test or two-way repeated measures ANOVA. The Mann-Whitney U test is used for non-normally distributed variables. Results were analyzed using SPSS version 26.0 (IBM, Armonk, NY, USA), with a significance level set at *P* < 0.05.

## Results

### Study population

Patient recruitment commenced in July 2022 and concluded in March 2023. Among the 150 patients assessed for eligibility, 26 were excluded, leaving a total of 124 patients that were randomly assigned. Following the allocation, both the experimental and control groups experienced six cases of participant loss. Ultimately, 112 participants (56 in the experimental group, 56 in the control group) were included in the analysis. All participants adhered to the prescribed intervention measures. Figure 1 illustrates the flow diagram according to the CONSORT guidelines, including reasons for participant dropout.

Table 1 presents the demographic and clinical characteristics of the patients, including gender, age, body mass index(BMI), diabetes, hypertension, ASA score, smoking history, alcohol intake history, tumor size, tumor differentiation grade, tumor location, surgical resection method, surgical approach, and preoperative clinical T and N staging. All baseline characteristics of patients in the IN and SEN groups were well-balanced.

**Table 1:** Baseline characteristics

Baseline characteristics	n	Group		*P*
		IN(n = 56)	SEN(n = 56)	
Gender				0.670
Male	82	40(71.4%)	42(75.0%)	
Female	30	16(28.6%)	14(25.0%)	
Age(years)	112	62.48 ± 10.207	60.77 ± 10.13	0.374
BMI(kg/m^2^)	112	23.75 ± 2.73	23.48 ± 3.09	0.624
Hypertension				0.801
No	93	46(82.1%)	47(83.9%)	
Yes	19	10(17.9%)	9(16.1%)	
DM				0.376
No	99	51(88.4%)	48(85.7%)	
Yes	13	5(8.9%)	8(14.3%)	
ASA grade				0.917
1	36	19(33.9%)	17(30.4%)	
2	64	31(55.4%)	33(58.9%)	
3	12	6(10.7%)	6(10.7%)	
Smoke				0.570
No	53	28(50.0%)	25(44.6%)	
Yes	59	28(50.0%)	31(55.4%)	
Drink				0.118
No	70	39(69.6%)	31(55.4%)	
Yes	42	17(30.4%)	25(44.6%)	
Tumor size(cm)		4.21 ± 2.319	4.12 ± 2.10	0.830
Differentiation of tumors				0.520
Low	96	46(82.1%)	50(89.3%)	
Middle	14	9(16.1%)	5(8.9%)	
High	2	1(1.8%)	1(1.8%)	
T stage				0.071
T1	22	12(21.4%)	10(17.9%)	
T2 T3	15 42	5(8.9%) 17(30.4%)	10(17.9%) 25(44.6%)	
T4	33	22(39.3%)	11(19.6%)	
N stage				0.149
N0	41	23(41.1%)	18(32.1%)	
N1	38	15(26.8%)	23(41.1%)	
N2	19	8(14.3%)	11(19.6%)	
N3	14	10(17.9%)	4(7.1%)	
Surgery, n(%)				0.319
Distal gastrectomy	87	38(67.9%)	45(80.4%)	
Proximal gastrectomy	16	10(17.9%)	6(10.7%)	
Total gastrectomy	13	8(14.3%)	5(8.9%)	
Surgical approach n(%)				0.327
Laparoscopy	71	38(67.9%)	33(58.9%)	
Robot	41	18(32.1%)	23(41.1%)	
HIPEC				0.447
No	62	29(51.8%)	33(58.9%)	
Yes	50	27(48.2%)	23(41.1%)	

BMI, body mass index; DM, diabetes mellitus; HIPEC, hyperthermic intraperitoneal peroperative chemotherapy

TNM stage according to the American Joint Committee on Cancer, 9th edition

### Postoperative infections and other complications

**Fig. 2:**
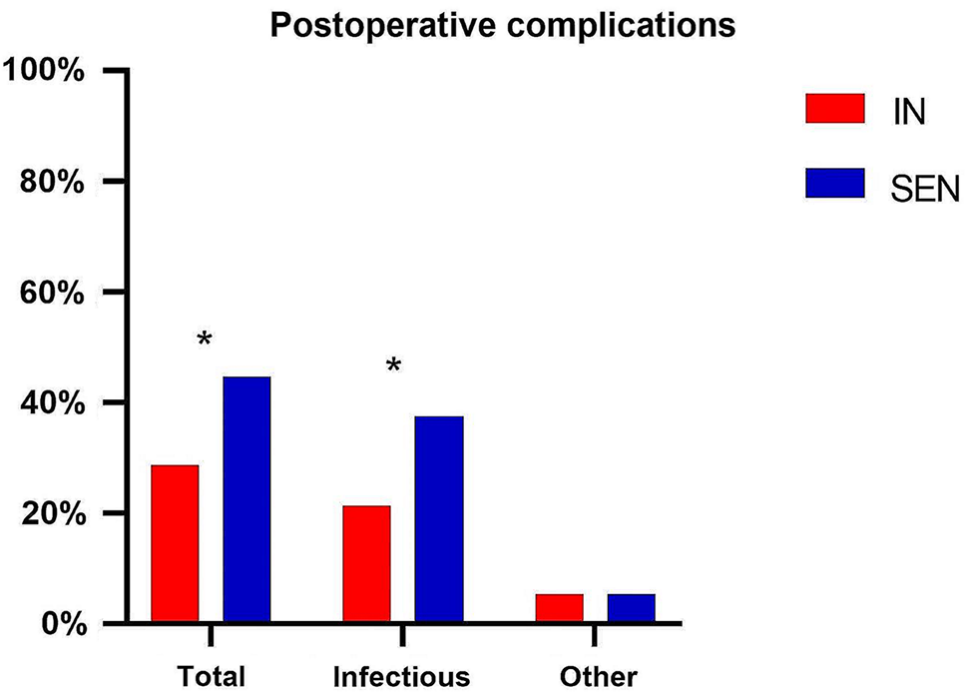
Incidence of postoperative complications. Comparison of total postoperative complications, infectious complications and non-infectious complications(*P < 0.05)

Table 2 present the results of postoperative complications. Both groups experienced gastrointestinal intolerance reactions during preoperative enteral nutrition support, but there was no significant difference between the two groups. Compared to patients in the SEN group, patients who received preoperative immunonutrition support had a significantly lower incidence of primary endpoint infectious complications (21.4% vs. 37.5%, *P* = 0.040)(Fig. 2). The most common infectious complication was pneumonia, with incidence rates of 23.2% in the SEN group and 12.5% in the IN group. There was no statistically significant difference in the incidence of postoperative non-infectious complications between the two groups. The overall incidence of postoperative complications (28.6% vs. 44.6%, *P* = 0.049) was significantly lower in the IN group compared to the SEN group, with a statistically significant difference between the two groups.Additionally, we classified the complications of both groups using the Clavien-Dindo classification. The results revealed no significant difference in the severity of postoperative complications between the two groups (*P* > 0.05).

**Table 2:** Postoperative infections and other complications

	IN(n?=?56)	SEN(n?=?56)	*P*
Gastrointestinal intolerance reactions, n(%)	5	2	0.242
Postoperative infectious complications, n(%)	12(21.4%)	22(37.5%)	0.040
Pneumonia	7(12.5%)	13(23.2%)	0.139
Urinary tract infection	4(7.1%)	7(12.5%)	0.508
Intraabdominal infection	1(1.8%)	2(3.6%)	0.558
CRBSI^*^	0	0	-
Other complication, n (%)	3(5.4%)	3(5.4%)	-
Pancreatic fistula	2(3.6%)	1(1.8%)	0.558
Gastroparesis	1(1.8%)	2(3.6%)	0.558
Surgical anastomotic leak	0	0	-
Total incidence of complication, n (%)	15(28.6%)	25(44.6%)	0.049
Clavien-Dindo classification, n (%)			
I- II	14(25.0%)	23(41.1%)	0.054
III	1(1.8%)	2(3.6%)	0.558

^*^CRBSI, Catheter-related bloodstream infection

### Postoperative laboratory inflammatory markers

**Fig. 3:**
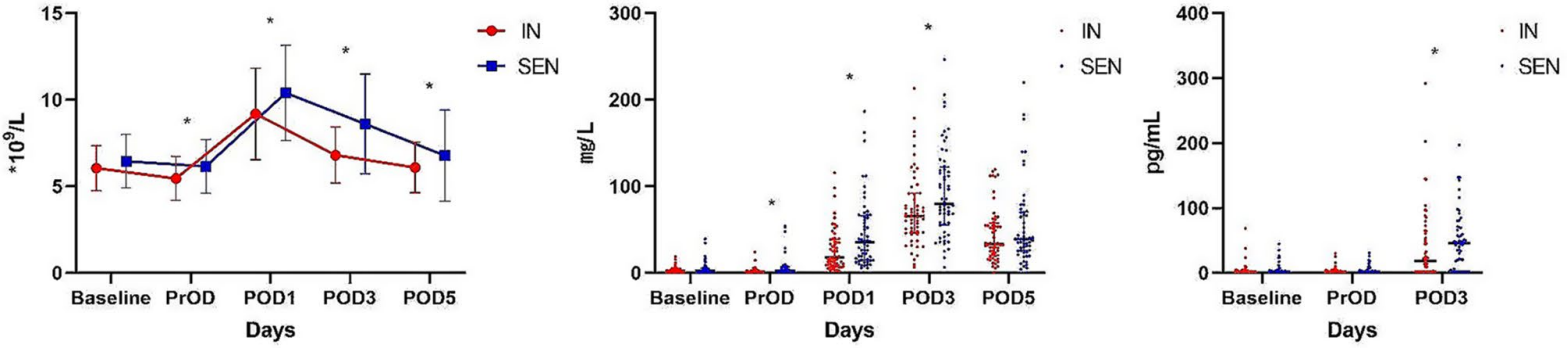
Postoperative laboratory inflammatory indexes. Postoperative laboratory inflammatory indexes(*P < 0.05). **(A)** WBC(×10^9^/L); **(B)** CRP(mg/L); **(C)** IL-6(pg/ml).WBC, white blood cell; CRP, C-reactive protein; IL-6, interleukin-6

Table 3; Fig. 3 demonstrate that preoperative administration of immunonutrition intervention leads to a reduction in patients’ laboratory inflammatory markers. The WBC levels were significantly lower in the IN group compared to the SEN group on the preoperative, postoperative day 1, postoperative day 3, and postoperative day 5 (*P* = 0.011;*P* = 0.019;*P* < 0.001;*P* = 0.034)(Fig. 3A). Moreover, by implementing preoperative immunonutrition intervention, the IN group exhibited significantly lower serum C-reactive protein (CRP) levels than the SEN group at preoperative, postoperative day 1, and postoperative day 3, with statistical significance (*P* = 0.005; *P* = 0.003; *P* = 0.042) (Fig. 3B). The IN group also displayed lower IL-6 levels on postoperative day 3 (*P* = 0.048)(Fig. 3C). However, there were no significant statistical differences observed between the two groups in other laboratory inflammatory markers such as procalcitonin (PCT), IL-1, IL-8, IFN-γ, and TNF-α.

**Table 3:** Postoperative laboratory inflammatory indexes

	IN(*n* = 56)	SEN(*n* = 56)	P
WBC(*10^9^/L) Baseline PrOD POD1 POD3 POD5	6.06 ± 1.30 5.46 ± 1.27 9.19 ± 2.64 6.81 ± 1.62 6.10 ± 1.45	6.46 ± 1.54 6.15 ± 1.55 10.40 ± 2.76 8.61 ± 2.88 6.78 ± 2.64	0.137 0.011 0.019 <0.001 0.034
CRP(mg/L) Baseline PrOD POD1 POD3 POD5	2.44(1.06–4.76) 1.19(0.51–3.05) 18.06(9.21–38.99) 65.83(46.40–92.30) 33.46(20.26–57.67)	2.25(0.94–5.35) 2.66(1.07–7.18) 35.99(15.10-66.23) 80.03(55.52-122.34) 39.18(25.78–70.41)	0.798 0.005 0.003 0.042 0.295
PCT(ng/mL) Baseline PrOD POD1 POD3 POD5	0.05(0.03–0.06) 0.04(0.03–0.06) 0.09(0.07–0.15) 0.12(0.08–0.18) 0.10(0.07–0.20)	0.05(0.02–0.06) 0.05(0.04–0.07) 0.08(0.05–0.12) 0.11(0.07–0.18) 0.09(0.06–0.15)	0.328 0.151 0.099 0.532 0.474
IL-1(pg/mL) Baseline PrOD POD3	1.70(1.55–5.78) 1.55(1.55–2.52) 1.55(1.55–1.79)	1.55(1.55–4.40) 1.55(1.55–1.79) 1.55(1.55–1.79)	0.530 0.506 0.839
IL-6(pg/mL) Baseline PrOD POD3	1.74(1.74–3.66) 1.74(1.74–3.15) 18.06(1.74–63.86)	1.74(1.74–3.99) 1.74(1.74–3.99) 45.97(9.43–69.97)	0.519 0.448 0.048
IL-8(pg/mL) Baseline PrOD POD3	1.89(1.89–43.46) 1.89(1.89–38.20) 1.89(1.89–5.32)	1.89(1.89–43.46) 1.89(1.89–43.46) 1.89(1.89–17.17)	0.621 0.921 0.925
IFN-γ(pg/mL) Baseline PrOD POD3	1.78(1.78-3.00) 1.78(1.78–3.02) 1.78(1.78–2.13)	2.08(1.78–3.20) 2.24(1.78–3.45) 1.78(1.78–1.78)	0.651 0.401 0.465
TNF-α(pg/mL) Baseline PrOD POD3	2.03(2.03–2.03) 2.03(2.03–2.03) 2.03(2.03–2.03)	2.03(2.03–2.03) 2.03(2.03–2.03) 2.03(2.03–2.03)	0.504 0.750 0.949

WBC, white blood cell count; CRP, C-reactive protein; PCT, procalcitonin; IL-1, 6, 8, interleukin-1, 6, 8 ; IFN-γ, interferon-gamma; TNF-α, tumor necrosis factor-alpha

### Postoperative laboratory immune markers

**Fig. 4:**
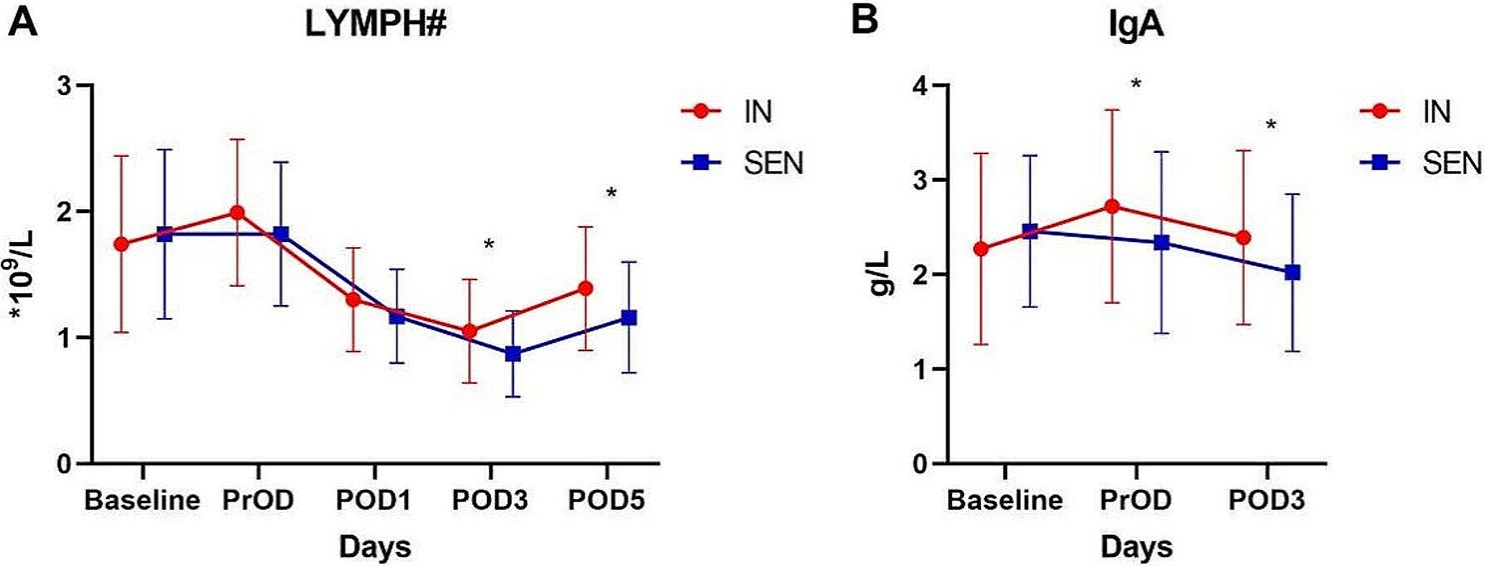
Postoperative laboratory immune indexes. Postoperative laboratory immune indexes (*P < 0.05). (**A**) LYMPH(×10^9^/L); (**B**) IgA(g/L). LYMPY, lymphocyte; IgA, immunoglobulin A.

As shown in Table 4; Fig. 4, preoperative administration of immunonutrition intervention can also enhance patients’ laboratory immune markers. The IN group exhibited significantly higher levels of blood lymphocytes than the SEN group on postoperative day 3 and postoperative day 5 (*P* = 0.016; *P* = 0.011), but no significant differences were observed at preoperative and postoperative day 1 (Fig. 4A). Additionally, this study monitored patients’ IgA levels and found that the IN group, after 7 days of preoperative immunonutrition intervention, showed significantly higher serum IgA levels compared to the SEN group at preoperative and postoperative day 3, with statistical significance (*P* = 0.048; *P* = 0.027) (Fig. 4B). However, no significant statistical differences were observed between the two groups in other laboratory immune markers such as CD4 T lymphocytes, CD8 lymphocytes, CD4/CD8 ratio, IgM, and IgG.

**Table 4:** Postoperative laboratory immune indexes

	IN(*n* = 56)	SEN(*n* = 56)	P
LYMPH#(*10^9^/L) Baseline PrOD POD1 POD3 POD5	1.74 ± 0.70 1.99 ± 0.58 1.30 ± 0.41 1.05 ± 0.41 1.39 ± 0.49	1.82 ± 0.67 1.82 ± 0.57 1.17 ± 0.37 0.87 ± 0.34 1.16 ± 0.44	0.530 0.126 0.078 0.016 0.011
CD4 + T cell(cells/µL) Baseline PrOD POD3	653.45 ± 259.44 677.09 ± 315.45 486.39 ± 197.90	706.41 ± 279.97 668.75 ± 263.52 452.21 ± 216.58	0.301 0.880 0.385
CD8 + T cell(cells/µL) Baseline PrOD POD3	363.82 ± 188.48 388.93 ± 212.28 264.43 ± 120.63	388.64 ± 120.75 364.80 ± 113.67 242.14 ± 102.29	0.408 0.455 0.294
CD4+/CD8+(cells/µL) Baseline PrOD POD3	2.37 ± 1.26 1.92 ± 0.66 1.92 ± 0.87	2.08 ± 0.78 2.07 ± 0.78 2.23 ± 1.18	0.141 0.279 0.113
IgA(g/L) Baseline PrOD POD3	2.27 ± 1.01 2.72 ± 1.02 2.39 ± 0.92	2.46 ± 0.80 2.34 ± 0.96 2.02 ± 0.83	0.258 0.048 0.027
IgM(g/L) Baseline PrOD POD3	1.08 ± 0.51 1.33 ± 0.58 0.90 ± 0.45	1.10 ± 0.65 1.20 ± 0.60 0.83 ± 0.46	0.853 0.253 0.377
IgG(g/L) Baseline PrOD POD3	12.34 ± 2.66 12.63 ± 2.48 10.02 ± 1.91	13.04 ± 3.04 11.95 ± 2.29 9.56 ± 2.42	0.197 0.133 0.268

LYMPH, lymphocytes; IgA, M, G, immunoglobulin A, M and G

### Postoperative recovery and other short-term clinical outcomes of this study

In addition to postoperative complications and infection-related laboratory markers, this study also examined the impact of preoperative administration of immunonutrition on other common short-term clinical outcomes, as shown in Table 5. We found that patients in the IN group had a shorter duration of antibiotic use, with statistical significance (*P* = 0.048), as the results mentioned above demonstrated a lower incidence of infectious complications in the IN group. Furthermore, due to fewer postoperative infections, patients in the IN group had a shorter length of hospital stay (*P* = 0.018) and lower total hospital costs compared to the SEN group (*P* = 0.034). However, in this study, no significant differences were observed between the two groups in terms of surgical duration and intraoperative blood loss. Additionally, the time to first flatus and bowel movement showed no significant differences, which are typically used to evaluate postoperative gastrointestinal functional recovery. One patient in the SEN group was readmitted within 30 days postoperatively due to delayed anastomotic fistula and intra-abdominal infection, requiring continuous irrigation with negative pressure using an infusion-style catheter. No deaths occurred within 30 days postoperatively in either group.

**Table 5:** Postoperative recovery and other short-term clinical outcomes

	IN(*n* = 56)	SEN(*n* = 56)	P
Surgical time(min), mean ± SD	208.13 ± 36.276	210.98 ± 51.50	0.735
Operative bleeding (mL), median (IQR)	20(10–50)	20(20–20)	0.659
Antibiotic usage, median (IQR)	1(1–4)	3(1–5)	0.048
Time to first flatus (h), median (IQR)	75(67–84)	77(69–89)	0.161
Time to first bowel movement (h), median (IQR)	97(89–108)	97(91–110)	0.262
Length of postoperative hospital stay(days)	8.89 ± 2.40	10.11 ± 2.90	0.018
Hospital costs(¥)	91785.76 ± 16874.70	99869.78 ± 22566.65	0.034
30-day readmission, n (%)	0(0%)	1(1.8%)	0.469
30-d mortality, n (%)	0(0%)	0(0%)	-

**Table 6:** Nutritive indexes

	IN(*n* = 56)	SEN(*n* = 56)	P
Weight loss	1.71 ± 1.48	2.25 ± 1.35	0.043
ALB Baseline PrOD POD1 POD3 POD5	39.73 ± 4.53 42.37 ± 4.70 34.90 ± 3.06 33.09 ± 3.40 33.96 ± 3.51	38.63 ± 3.92 41.33 ± 4.10 34.71 ± 3.85 32.79 ± 3.08 33.73 ± 3.02	0.171 0.213 0.774 0.628 0.712
PAB Baseline PrOD POD1 POD3 POD5	265.83 ± 58.33 281.21 ± 56.83 208.98 ± 49.35 146.99 ± 34.75 152.96 ± 44.67	261.48 ± 53.23 275.09 ± 49.11 207.53 ± 49.44 149.78 ± 41.55 159.18 ± 47.66	0.681 0.544 0.877 0.329 0.478
HB Baseline PrOD POD1 POD3 POD5	127.55 ± 22.75 134.23 ± 22.32 120.27 ± 21.49 115.95 ± 18.27 113.75 ± 17.63	124.41 ± 22.85 131.05 ± 23.77 121.68 ± 20.32 114.93 ± 18.36 114.80 ± 17.25	0.467 0.477 0.722 0.769 0.750

ALB, serum albumin;PAB, prealbumin;HB, hemoglobin

### Nutritional status and nutritional markers

Patients in the IN group experienced an average weight loss of 1.71 kg from preoperative to postoperative day 5, while the SEN group had an average weight loss of 2.25 kg. The IN group exhibited significantly less weight loss during this period, with statistical significance (*P* = 0.043). However, no significant differences were observed between the two groups in terms of albumin, prealbumin, and hemoglobin levels.

## Discussion

The results of this study demonstrate that preoperative application of immunonutrition can improve short-term clinical outcomes in gastric cancer patients with cachexia, including reducing the incidence of postoperative infectious complications, improving inflammatory and immune markers, attenuating perioperative weight loss, ultimately shortening postoperative hospital stay, decreasing antibiotic usage, and reducing healthcare costs.

The question of whether preoperative use of immunonutritional preparations in gastric cancer patients can reduce postoperative complications, especially infectious complications, has been a topic of considerable debate. A recent meta-analysis reported a significant impact of preoperative immunonutrition on reducing the incidence of postoperative infectious complications following major abdominal surgery [27, 28]. However, the included studies had notable limitations, such as small sample sizes [29–31] and conflicts of interest [32, 33], which led to bias and restricted the generalizability of the research findings. The findings of this study indicate that preoperative administration of immunonutrition formulations can reduce the incidence of postoperative infectious complications and overall complications in gastric cancer cachexia patients. This could be attributed to the specific inclusion of the cachexia condition as a limiting factor within our study cohort. Cancer cachexia patients typically exhibit more severe malnutrition, inflammation, and immunodeficiency. In addition to providing energy, immunonutrition comprising substances such as omega-3 fatty acids, arginine, and nucleotides can regulate inflammation, cellular immune function, and stress response in critically ill patients. Omega-3 fatty acids are derived from fish oil and exert anti-inflammatory and immune-modulating effects by regulating the synthesis of various eicosanoids [34]. Preoperative and postoperative administration of a diet rich in omega-3 fatty acids can reduce levels of eicosanoids, including leukotrienes, specific leukotrienes, thromboxanes, and prostaglandins, in plasma and tissues, all of which have pro-inflammatory and immune-suppressive effects [35–37]. Arginine is considered a trigger for T cells, which proliferate in response to mitogens or cytokine stimulation [38]. This is one well-known role of arginine in immune cells, some of which are mediated through the L-arginine-nitric oxide (NO) pathway [38, 39]. However, in early-stage gastric cancer patients, the influence of immune-modulating nutrients may be overshadowed, particularly in contrast to cachectic cancer patients who experience severe malnutrition and a heightened inflammatory state. Therefore, the effect of reducing postoperative complications is more significant in cachectic gastric cancer patients [40, 41]. Due to the reduction in postoperative infectious complications and overall complications, the IN group exhibits shorter duration of antibiotic use and hospital stay, as well as lower hospital costs compared to the control group.

TNF-α, IL-6, and CRP play important roles in early tissue injury and inflammatory response following trauma [42, 43]. TNF-α and IL-6 are pro-inflammatory cytokines and have crucial roles in the induction and regulation of inflammation [8]. TNF-α, produced by lipopolysaccharide-stimulated monocytes and macrophages, acts as a pleiotropic initiator of inflammation. TNF-α can activate neutrophils, macrophages, and other inflammatory cells, as well as induce IL-6 secretion by endothelial cells. IL-6 is an important marker reflecting the severity of inflammation and tissue damage [12]. Our study results showed a significant difference in IL-6 levels on the third postoperative day between the immunonutrition (IN) group and the control group. Although the TNF-α levels were lower in the IN group on both preoperative and the third postoperative day, the difference did not reach statistical significance. CRP is an acute-phase protein synthesized by the liver under the induction of IL-6. CRP levels demonstrate rapid and sensitive changes in acute trauma and infection, thus reflecting alterations in the body’s inflammatory response. Continuous monitoring of CRP postoperatively is a sensitive indicator to assess the degree of postoperative stress response and the development of infectious complications, which holds important clinical significance. The present study have shown that preoperative immunonutrition intervention significantly reduced CRP levels on the first and third postoperative day in the IN group compared to the control group, suggesting that immunonutrition can alleviate the inflammatory response in patients with gastric cancer and malnutrition following surgery.

The research conducted by Braga et al. demonstrates that the perioperative administration of immunonutrition can prevent early postoperative impairment of phagocytic function, delay hypersensitivity reactions, and increase the total lymphocyte count [44]. The present study confirms the aforementioned findings, as the results indicate that the experimental group, following 7 days of enteral immunonutrition intervention, exhibited a slower decline in postoperative total lymphocyte count compared to the control group, with significant differences observed on the third and fifth postoperative days.T lymphocyte-mediated cellular immunity plays a crucial role in anti-tumor immune response [45, 46]. Subsets of T lymphocytes, including CD4^+^, CD8^+^, and CD4^+^/CD8^+^ ratio, serve as sensitive indicators of cellular immune function. CD4^+^ T cells promote B cell differentiation (inducing antibody production) and activate other cells to secrete lymphokines, exerting a mediating role in inflammatory reactions. CD8^+^ T cells function as immunosuppressive cells, inhibiting antibody secretion and T cell proliferation, and may represent cytotoxic cells [47]. Meta-analytical findings suggest that omega-3 polyunsaturated fatty acids can enhance postoperative cellular immune function in patients with gastrointestinal malignancies. Studies have indicated that in the initial week following surgery, patients experience a decrease in blood CD4^+^ levels, a decreased CD4^+^/CD8^+^ ratio, and an elevated CD8 + levels, suggesting suppression of cellular immune function. The ratio alteration resulting from surgical trauma and postoperative metabolic stress hampers T lymphocyte functionality, consequently diminishing both cell abundance and immune response intensity [48, 49]. However, no significant differences were observed between the two groups in our study, the T lymphocyte count and immune response intensity of the experimental group patients did not show any improvement as a result of the utilization of immunonutrition. This study demonstrates significant differences in IgA levels between the IN group and the control group prior to surgery and on the third day after surgery. It is cautiously inferred that preoperative use of immunonutrition may improve humoral immune function markers.Furthermore, there is evidence suggesting that preoperative utilization of immunonutrition can enhance the degree of postoperative weight loss improvement in patients, a result validated in this study [50, 51]. The experimental group exhibited ameliorated postoperative weight loss compared to the control group. However, no disparities were observed in nutritional indicators such as ALB and PAB.

The limitations of this study are as follows. 1.Although surgeons, radiologists, pathologists, and data managers were unaware of the interventions received by the patients, blinding of the patients was not achieved. We provided comprehensive information to the patients prior to the commencement of the trial and closely monitored and followed up on their compliance with the interventions during the process. However, bias may still be present; 2.We had dedicated personnel conducting phone follow-ups for seven days before the surgery, but we cannot guarantee patient compliance with the use of nutritional supplements, which may impact the results of the study;3. Since this study was conducted at a single institution in China, there may be issues with external validity.Further large-scale, multi-center studies should be conducted to validate the findings and determine their generalizability to Western populations or other populations. However, the current study has the advantages of a well-designed study and a randomized controlled trial initiated by the researchers. This randomized controlled trial provides a more solid foundation for determining whether preoperative immunonutrition should be given to gastric cancer patients with malnutrition.

## Conclusion

Preoperative administration of immunonutrition formula has a positive impact on the incidence of infectious complications in malnourished gastric cancer patients after surgery. It improves patients’ inflammatory and immune status, shortens hospital stays, and reduces healthcare costs. Preoperative use of immunonutrition may contribute to the improvement of prognosis in this high-risk population.

### Electronic supplementary material

Below is the link to the electronic supplementary material.

Supplementary Material 1

## Data Availability

No datasets were generated or analysed during the current study.

## Declarations

### Statement

The data used to support the findings of this study may be released upon application to the Department of Gastrointestinal Surgery, The Affiliated Hospital of Qingdao University, who can be contacted at shougencao@qdu.edu.cn.
