# Supplementary material for: Effect of preoperative immunonutrition on postoperative short-term clinical outcomes in patients with gastric cancer cachexia: a prospective randomized controlled trial
Source: World J Surg Oncol. 2024 Apr 17;22:101. doi: 10.1186/s12957-024-03348-y (PMC11022452; doi:10.1186/s12957-024-03348-y)
Supplement: Supplementary file 1 — Supplementary Material 1 [file 12957_2024_3348_MOESM1_ESM.pdf]

附件 2

国家市场监督管理总局
特殊医学用途配方食品产品标签、说明书

国食注字 TY20220004

特殊医学用途肿瘤全营养配方食品

速熠素

【产品类别】特定全营养配方食品

【配料表】水、麦芽糊精、酪蛋白酸钠、酪蛋白酸钙、鱼油、中链甘油三酯、植物油（低芥酸菜籽油，葵花籽油）、白砂糖、醋酸维生素 A、胆钙化醇、dl-α-醋酸生育酚、植物甲萘醌、盐酸硫胺素、核黄素、盐酸吡哆醇、氰钴胺、烟酰胺、叶酸、D-泛酸钙、L-抗坏血酸钠、D-生物素、柠檬酸钠、氯化钠、磷酸氢二钠、柠檬酸钾、氯化钾、硫酸铜、氧化镁、硫酸亚铁、硫酸锌、硫酸锰、磷酸二钙、碘化钾、亚硒酸钠、氯化铬、钼酸钠、酒石酸氢胆碱、5'单磷酸胞苷、5'单磷酸腺苷、5'-鸟苷酸二钠、5'-尿苷酸二钠、半乳甘露聚糖、L-精氨酸、柠檬酸、单，双甘油脂肪酸酯、食用香精、磷脂、微晶纤维素、羧甲基纤维素钠、卡拉胶、β-胡萝卜素。

【营养成分表】

营养成分	每 100mL	每 100kJ
能量(kJ)	593.35	100
蛋白质(g)	8.6	1.45
脂肪(g)	5.4	0.91
n-3 脂肪酸（以 EPA 和 DHA 计）供能比(%)	2.5	2.5
二十二碳六烯酸（DHA）(mg)	157	26.5
二十碳五烯酸（EPA）(mg)	243	40.95
碳水化合物(g)	14.1	2.38
维生素 A(μg RE)	102.3	17.25

No. 22004119

营养成分	每 100mL	每 100kJ
维生素 D(μg)	1.6	0.27
维生素 E(mg α-TE)	7.1	1.2
维生素 K_1(μg)	8.3	1.4
维生素 B_1(mg)	0.16	0.03
维生素 B_2(mg)	0.22	0.04
维生素 B_6(mg)	0.23	0.04
维生素 B_{12}(μg)	0.6	0.10
烟酸(mg)	0.8	0.13
叶酸(μg)	35.5	5.98
泛酸(mg)	1.48	0.25
维生素 C(mg)	88	14.83
生物素(μg)	7.1	1.2
钠(mg)	145	24.44
钾(mg)	195	32.86
铜(μg)	130	21.91
镁(mg)	34	5.73
铁(mg)	1.8	0.3
锌(mg)	1.6	0.27
锰(μg)	400	67.41
钙(mg)	100	16.85
磷(mg)	85	14.33
碘(μg)	16	2.7
氯(mg)	140	23.59
硒(μg)	6	1.01
铬(μg)	7.7	1.3
钼(μg)	11.8	1.99
胆碱(mg)	59.2	9.98

[illegible]巢健康科学（中国）有限公司
NESTLE HEALTH SCIENCE (CHINA) LTD.

No. 22004120

营养成分	每 100mL	每 100kJ
核苷酸(mg)	180.6	30.44
膳食纤维(g)	0.9	0.15
精氨酸(g)	1.7	0.29

【配方特点/营养学特征】本品为特殊医学用途肿瘤全营养配方食品，能量密度约为1.4kcal/mL。每瓶（250mL）提供能量约 354kcal，约含蛋白质 21.5g、核苷酸 451.5mg、精氨酸 4.25g、n-3 脂肪酸（以 EPA 和 DHA 计）1g。添加了膳食纤维，以半乳甘露聚糖作为来源。本品可作为单一或部分营养来源满足肿瘤患者营养需求。

【临床试验】本产品开展了一项前瞻性、多中心、随机对照、平行开放的临床研究，共入组257 例围手术期或行肿瘤切除术的消化道肿瘤患者，试验周期为术前 5 天至术后第 8 天，随访至术后 30 天。本研究以已批准的相应类别肠内营养制剂为对照，其中"术后第 8 天与基线相比血清前白蛋白的水平变化"的研究结果为试验组不劣于对照组；两组在生命体征、生化指标、胃肠道耐受性、握力、ECOG 体力状况评分、白蛋白、IL-6 水平、CD4+/ CD8+、C反应蛋白等的差异无统计学意义。

【组织状态】液态

【适用人群】10 岁以上存在营养风险或营养不良的肿瘤患者

【食用方法和食用量】口服或管饲。食用方法和食用量应由医生或临床营养师根据适用人群的年龄、体重、医学状况等综合确定。

【净含量和规格】250mL

【保质期】12 个月

【贮存条件】未开封的产品在常温干燥处贮存，开封后的产品需盖紧存放在冰箱中冷藏（0~4℃），并在 24 小时内食用完。

【警示说明和注意事项】

1.请在医生或临床营养师指导下使用。

2.本品禁止用于肠外营养支持和静脉注射。

3.建议使用本品前进行营养筛查和（或）营养评估。

4.不适用于非目标人群使用。

5.不适用于严重脓毒症等患者。

6.不适于应用肠内营养的患者禁用本品。

No. 22004121

7.对本品中所含物质过敏的人群禁用。

8.用于管饲时，禁止在本品中添加其他食物和药物。

9.本品渗透压约为 480mOsmol/L，供临床使用参考。

10.本包装不能用于微波加热。

11.若有少量沉淀物属于正常现象，使用前请摇匀。

12.使用不当可能引起健康危害。

雀巢健康科学（中国）有限公司

注：企业上市产品的标签、说明书，还应当符合相关法律、法规、规章和食品安全国家标准的规定。

No. 22004122
